# Reevaluation of ambiguous genetic variants in sudden unexplained deaths of a young cohort

**DOI:** 10.1007/s00414-023-02951-0

**Published:** 2023-01-25

**Authors:** Estefanía Martinez-Barrios, Georgia Sarquella-Brugada, Alexandra Perez-Serra, Anna Fernandez-Falgueras, Sergi Cesar, Mireia Alcalde, Mónica Coll, Marta Puigmulé, Anna Iglesias, Carles Ferrer-Costa, Bernat del Olmo, Ferran Picó, Laura Lopez, Victoria Fiol, José Cruzalegui, Clara Hernandez, Elena Arbelo, Nuria Díez-Escuté, Patricia Cerralbo, Simone Grassi, Antonio Oliva, Rocío Toro, Josep Brugada, Ramon Brugada, Oscar Campuzano

**Affiliations:** 1 Pediatric Arrhythmias, Inherited Cardiac Diseases and Sudden Death Unit, Cardiology Department, Sant Joan de Déu Hospital de Barcelona, 08950 Barcelona, Spain; 2 Low Prevalence and Complex Diseases of the Heart (ERN GUARD-Heart), European Reference Network for Rare, 1105 AZ Amsterdam, The Netherlands; 3 Malalties Cardiovasculars en el Desenvolupament, Institut de Recerca Sant Joan de Déu, Arrítmies Pediàtriques, Cardiologia Genètica i Mort Sobtada, Esplugues de Llobregat, 08950 Barcelona, Spain; 4 Medical Science Department, School of Medicine, University of Girona, 17003 Girona, Spain; 5 Cardiovascular Genetics Center, University of Girona-IDIBGI, 17190 Girona, Spain; 6 Centro de Investigación Biomédica en Red. Enfermedades Cardiovasculares (CIBERCV), 28029 Madrid, Spain; 7 Arrhythmias Unit, Hospital Clinic, University of Barcelona-IDIBAPS, 08036 Barcelona, Spain; 8 Department of Health Sciences, Section of Forensic Medical Sciences, University of Florence, Largo Brambilla 3, 50134 Florence, Italy; 9 Department of Health Surveillance and Bioethics, Section of Legal Medicine, Fondazione Policlinico A. Gemelli IRCCS, Università Cattolica del Sacro Cuore, 00168 Rome, Italy; 10 Medicine Department, School of Medicine, 11003 Cadiz, Spain; 11 Cardiology Service, Hospital Josep Trueta, University of Girona, 17007 Girona, Spain

**Keywords:** Sudden unexplained death, Infant, Pediatric, Genetics, Interpretation

## Abstract

Sudden death cases in the young population remain without a conclusive cause of decease in almost 40% of cases. In these situations, cardiac arrhythmia of genetic origin is suspected as the most plausible cause of death. Molecular autopsy may reveal a genetic defect in up to 20% of families. Most than 80% of rare variants remain classified with an ambiguous role, impeding a useful clinical translation. Our aim was to update rare variants originally classified as of unknown significance to clarify their role. Our cohort included fifty-one post-mortem samples of young cases who died suddenly and without a definite cause of death. Five years ago, molecular autopsy identified at least one rare genetic alteration classified then as ambiguous following the American College of Medical Genetics and Genomics’ recommendations. We have reclassified the same rare variants including novel data. About 10% of ambiguous variants change to benign/likely benign mainly because of improved population frequencies. Excluding cases who died before one year of age, almost 21% of rare ambiguous variants change to benign/likely benign. This fact makes it important to discard these rare variants as a cause of sudden unexplained death, avoiding anxiety in relatives’ carriers. Twenty-five percent of the remaining variants show a tendency to suspicious deleterious role, highlighting clinical follow-up of carriers. Periodical reclassification of rare variants originally classified as ambiguous is crucial, at least updating frequencies every 5 years. This action aids to increase accuracy to enable and conclude a cause of death as well as translation into the clinic.

**Supplementary Information:**

The online version contains supplementary material available at 10.1007/s00414-023-02951-0.

## Introduction 

Sudden unexpected death in young population (SUDY) is a rare event but provokes an awful familiar and social impact due to patients had remained without symptoms during their lifetime [1]. Nowadays, this lethal event remains a current challenge both in the clinical field because decease is the first manifestation of an undetected arrhythmogenic disease and in the forensic area due to large part of cases remain unresolved after a comprehensive medico-legal autopsy. In nearly 40% of cases SUDY cases less than 18 years old, no structural alterations are identified [2]. SUDY cohort did not include cases less than 1 year of age died suddenly, [3], named sudden infant death syndrome (SIDS). Lethal episode in SIDS is considered multifactorial and the pathophysiological mechanism underlying the death is still unknown in large part of cases [4]. Cases in which neither macroscopic nor microscopic heart impairments are found at autopsy can be defined as sudden unexplained death (SUD), and in these cases, post-mortem genetic testing should be indicated in order to exclude an inherited arrhythmic entity [5–9]. Pathogenic alterations in genes associated with cardiomyopathies have been also suggested as potential cause of SUD, inducing first a malignant arrhythmogenic event than structural alteration [10], especially in infants/children [11]. As a consequence, molecular autopsy is recommended in SUDY/SIDS cases [12], which is focused on unraveling the genetic cause of sudden death but also has crucial implications both for diagnosis and adoption of preventive measures in victims’ relatives at risk [13]. Despite this widely accepted fact, its routine application is not yet performed in forensic medicine [14].

Next-generation sequencing (NGS) technologies have emerged as a cost-effective approach focused on analyzing a wide spectrum of genes in a reduced time, increasing the diagnostic yield [15, 16]. However, a large part of rare variants generated remains classified of unknown significance (VUS) due to lack of data, impeding a proper interpretation, and useful clinical translation [17]. The current American College of Medical Genetics and Genomics (ACMG) recommendations focused on the classification of genetic variants which state that the role of a variant may change depending on the data available despite no suggestion concerning reclassification was included [18]. Re-analyzing the same genetic data with the context provided by new improvements may resolve cases previously remaining unsolved to date, possibly leading to significant benefits to the family and health care system [19]. Recent studies focused on the reanalysis of rare variants associated with inherited arrhythmogenic syndromes (IAS), highlighting that 20–30% of variants modify their previous role [20–24], increasing the percentage if firstly classified not following ACMG recommendations [25]. Modification of role principally occurs in rare variants classified as VUS and mainly due to update on population frequencies [22, 23]. To date, only one study has been focused on the re-evaluation of variants not following ACMG recommendations in SUD cases [26]. Therefore, no research study has been reported concerning the reclassification of rare variants associated with SUD in the young population, so far. In our study, we reanalyzed a young cohort of post-mortem cases to investigate the current role of rare variants classified as VUS 5 years ago.

## Materials and methods

### Study cohort

Our retrospective study included fifty-one post-mortem cases who died suddenly at young age (< 17 years old) with a no definitive cause of death after a complete medico-legal autopsy. All samples were analyzed in our laboratory between 2015 and 2017. After comprehensive genetic analysis using an NGS approach, all patients had an inconclusive genetic diagnosis explaining the origin of the disease. All patients carried at least a rare variant, and all variants were classified as having an unknown/ambiguous role according to ACMG recommendations [18]. Cases with a doubtful inconclusive post-mortem diagnosis were not included in the present study to avoid bias. All cases carrying a rare variant in any of the genes analyzed and classified as definite or potentially causative of disease were not included following the same approach. Genetic analysis was approved by the ethics committee of Hospital Josep Trueta (Girona, Catalonia, Spain) following the World Medical Association Declaration of Helsinki. Available clinical and genetic data on all patients were anonymized and kept confidential. Consent was obtained from the judge (including in current law of our country) before genetic analysis.

### Genetic analysis

Suppl.1

### Data sources

Suppl.2

### Classification/interpretation

All rare variants were classified between 2015 and 2017, always following ACMG recommendations [18]. Currently, all rare variants are classified following the same guidelines but including updates; the PM2 item in the ACMG recommendations was considered fulfilled if the MAF in relevant population databases was ≤ 0.01% [27]. Except HCM (1/500, MAF 0.2%), all IAS are rare diseases (less than 1/2000, being MAF less than 0.05%). In addition, the vast majority of reported pathogenic variants in IASs are very rare (MAF < 0.005%) [28], consistent with all variants currently classified as likely pathogenic/pathogenic (LP/P), showing an extremely rare frequency (MAF < 0.001%) (www.gnomad.broadinstitute.org/ and www.clinicalgenome.org/). Variants classified by 2022 as VUS were subclassified as VUS-likely benign (VUS-LB) (MAF > 0.001%, and no definite IAS association) and VUS-LP (not MAF or MAF < 0.001% and certain IAS association). We included genes definitively associated with cardiac channelopathies and cardiomyopathies [12]. These subgroups were studied to clarify their potential ambiguous role in clinical practice. Genetic data were independently evaluated and classified by five authors (EMB, GSB, EA, RB, and OC), specialists in the genetics of inherited arrhythmias field, to avoid bias. All investigators agreed on a final classification of all rare variants included in this study.

## Results

### Global cohort

Our retrospective study included fifty-one cases (of which 64.7% were males) of young age (ages ranging from one month to sixteen years), all Caucasians who died suddenly, and whose dead were classified as unexplained after a complete medico-legal autopsy. Review of forensic data did not change the definite diagnostic from 5 years ago in any case. It is widely accepted that sudden death in first year of life is a controversial event and considered as an entity per se SIDS [4]. Therefore, we have divided the whole cohort in two groups (less than 1 year of age and from 1 to 16 years old, young cohort) (ESP_Tab1, ESP_Tab2, ESP_Fig1).

### Reinterpretation

Concerning whole cohort, reanalysis including all current available data and following ACMG recommendations showed that six (10.52%) rare variants previously classified as VUS downgraded deleterious role to LB (four variants, 7.02%) and B (two variants, 3.5%). None of the rare variants upgraded to LP or P. Importantly, all modifications of the first classification to LB and B were because of increased MAF from the previous classification to the present (ESP_Tab1, ESP_Tab2, ESP_Fig1).

In the SIDS cohort, the reanalysis showed that two (5.26%) rare variants previously classified as VUS downgraded deleterious role to B (two variants), both because of increased MAF from the previous classification to the present. None of the rare variants upgraded to LP or P. Concerning MAF, eighteen (47.36%) variants did not show any population frequency in the present analysis, in contrast to twenty-nine in 2017 (76.31%). In addition, five (13.15%) variants showed nowadays MAF < 0.001%, similar to four (10.52%) in 2017. In VUS group including thirty-six rare variants, nine variants (25%) were reclassified as VUS-LB and ten (27.77%) as VUS-LP. Concerning genes encoding cardiac ion channels/associated proteins of sixteen rare variants, one (6.25%) decreases ambiguity to B. Of fifteen rare variants still classified as VUS, ten variants (66.66%) were reclassified as VUS-LP and five to VUS-LB. Concerning genes encoding structural proteins of myocyte, of twenty-two rare variants, one decreases ambiguity to B (4.54%). Of twenty-one rare variants still classified as VUS, thirteen (61.9%) were reclassified as VUS-LP, and eight variants (38.09%) were reclassified as VUS-LB (ESP_Tab1, ESP_Tab2, ESP_Fig1).

In the young cohort (excluding SIDS cases), the reanalysis showed that four of nineteen (21.05%) rare variants previously classified as VUS downgraded deleterious role to LB, most part due to an increased MAF from the previous classification to the present. None of the rare variants upgraded to LP or P. Concerning MAF, seven (36.84%) variants did not show any population frequency in the present analysis, in contrast to fourteen in 2017 (73.68%). In addition, only one (5.26%) variant showed nowadays MAF < 0.001%, but none in 2017. In fifteen VUS variants, six variants (40%) were reclassified as VUS-LB and three to VUS-LP (20%). Concerning seven variants in genes encoding cardiac ion channels/associated proteins, three variants were reclassified to LB (42.85%). Of four rare variants classified as VUS, one was considered VUS-LB (25%) and two variants as VUS-LP (50%). Concerning twelve variants in genes encoding structural proteins of myocyte, one decreases ambiguity to LB (8.33%). Of eleven rare variants still remain classified as VUS nowadays, five variants (45.45%) were reclassified as VUS-LB and one to VUS-LP (9.09%) (ESP_Tab1, ESP_Tab2, ESP_Fig1).

## Discussion

Improvements of available clinical forensic and/or genetic data may modify a previous classification of rare variants remaining classified as VUS, highlighting the importance of periodically reanalysis [29]. This fact is crucial for identifying the cause of SUDY/SIDS but also for surviving relatives due to early identification of a definite deleterious alteration which could tailor the therapeutic management, reducing the risk of malignant arrhythmias [30]. Our study analyzed, for the first time, a post-mortem cohort of cases less than 17 years of age who died suddenly, without explanation after comprehensive medico-legal and molecular autopsy.

Our reanalysis shows that more than 10% of rare variants classified as VUS 5 years ago now decrease its potential deleterious role as cause of unexplained death in our young post-mortem cohort. Therefore, these variants can be a priori overlooked as the main cause of unexplained death helping in a more accurate genetic counseling in their relatives [19], mainly for asymptomatic relatives who carry the same genetic alteration. However, in symptomatic genetic carriers, no interpretation should be made on its possible role in phenotype modification, and no influence on clinical management should be adopted [12, 30, 31]. At this point is important to remark that disease-specific phenotypes significantly increase the accuracy of classification and reinforce the need for clinical data in genetic diagnoses, aiding in VUS interpretation [21]. In addition, family segregation (genotype–phenotype correlation, if possible, at least 3 generations) is also crucial to clarify the potential role of a rare variant classified as VUS following ACMG recommendations. None of both approaches is included in post-mortem cohort analyzed in our study, thus reducing percentage of VUS variants with a conclusive role after reevaluation. Therefore, the main update which helps to reclassify VUS variants in our cohort is population frequencies.

From a medico-legal point of view, a periodic reanalysis of rare variants classified as VUS should be considered as a standard of care for molecular autopsy, and failure to comply with this best practice could lead to accusation of malpractice and to liability for missed diagnosis or harmful interventions due to misdiagnosis [32]. This genetic reanalysis and clinical-forensic reinterpretation should be performed in a personalized way [33], despite no time period of reanalysis is state to date. Previous studies focused on IAS suggested to perform a reanalysis if variants not currently classified following ACMG recommendations [20, 21, 24, 25, 29, 34], and from 2 to 5 years if following ACMG recommendations [23, 35, 36]. Concerning SUDY/SIDS, our study suggests maximum of 5 years as suitable time period of reanalysis, at least including an update on population frequencies; this item is the main item responsible for most part of modifications in our cohort, in concordance to previous studies [23]. Finally, as also occurs in previous studies concerning reclassification in IAS [23, 25], the economic cost of a comprehensive reinterpretation and who should perform and assume this cost is not assessed in our study. This remains a controversial point that should be deeply analyzed.

Due to high % of variants remaining as VUS, we have moved one step more to shed light on the uncertainty implicit to rare variants which remain currently classified as VUS, as recently performed by our group in IAS [37]. At this point, it is crucial to highlight that our approach is suggestive that no clinical translation and implementation/modification of therapeutic measures should be performed according to our suggested categorization. Hence, of fifty-one rare variants that remain as VUS after reclassification nowadays, nearly 55% show but do not confirm, a tendency to clarify their role (29.41% to VUS-LB and 25.49% to VUS-LP). Finally, our VUS variants’ trend to change show that the main parts (23.52% of 25.49%, 92.3%), increasing the potential deleterious role (VUS-LP), are located in genes encoding cardiac ion channels or associated proteins; in contrast, main parts (27.45% of 29.41%, 93.33%) of VUS variants showing a tendency to decrease pathogenicity (VUS-LB) are located on genes encoding structural proteins of myocyte. In addition, only 5.88% VUS variants can be suggested as VUS-LP in young cohort and are similarly distributed in genes related to channelopathies or cardiomyopathies (ratio 2:1 respectively). In contrast, large part (19.6% of 23.52%, 83.33%) of VUS-LP variants was identified in SIDS cohort. This fact is according to widely accepted association of deleterious alterations in cardiac ion channel as main responsible of SIDS [38].

In SUDY/SIDS cohorts, the role of rare variants in genes encoding structural proteins of myocyte still remains controversial [11], highlighting the entity of “concealed cardiomyopathy” [10, 39–41]. However, to avoid diagnostic miscues in cases without overt phenotypes, as well as ambiguity in victims’ relatives, a careful genetic interpretation is vital before clinical translation [42, 43]. In our cohort, three patients harbored a rare deleterious variant in genes encoding structural proteins (*PKP2*_p.Arg413Ter, *MYH7*_p.Arg671Cys, and *TNNI3_*p.Arg204His). In the case carrying *PKP2*_p.Arg413Ter, a myocarditis was identified after autopsy, suggesting a genetic predisposition to arrhythmias and being myocarditis the most plausible trigger [44]. In the second case, no structural alteration was identified, and in the third case, who carried *TNNI3_*p.Arg204His, a slight fibrosis in both ventricles was detected, suggesting a first stages of any cardiomyopathy but not with a conclusive diagnosis after histological analysis. Although a purely genetic classification of these variants as deleterious in structural pathology was clear, the definitive role of these genetic changes to be responsible for sudden death in our patients is controversial due to lack of previous definite clinical diagnose of cardiomyopathy or any structural alteration suggesting a potential first stage of cardiomyopathy. At this point, it could be firstly remarking the necessity of a purely genetic classification of rare variants in IAS, only focused on genetic data (mainly type of alteration, population frequency, functional studies, in silico predictions) in contrast to the widely called genetic diagnosis which may include genetic data abovementioned but also clinical, familial, and forensic information in order to perform a proper translation of genetic results into clinical practice.

Limitations: Firstly, a lack of previous clinical diagnosis in deceased cases impedes a conclusive correlation with any potential causative gene. Secondly, a lack of available functional data for mostly of rare variants impedes a more accurate interpretation. Thirdly, there is a lack of family segregation, which is critical to clarify the role of a rare variant in SUDY/SIDS cases. Finally, cases included in our cohort may carry additional rare variants in other genes not included in our panel.

## Conclusions

Genetic analysis in SCD-related diseases obtains rare variants potentially deleterious, but lack of data impedes a proper variant classification, leading to many variants currently being classified as VUS. Updating genetic data following ACMG recommendations change nearly 10% of rare missense variants that were classified 5 years ago with an ambiguous role. This percentage of rare variants is currently considered without a deleterious role, mainly because of the high increase in population frequencies. It helps to discard these variants as causative in relatives, reducing anxiety to be carrier of a rare variant without an ambiguous role. Our results support periodic reclassification of variants previously classified as VUS, at least concerning frequencies, suggesting no more than 5-year time limit. In forensic field, a low rate of VUS gets a definitively deleterious role mainly due to lack of additional clinical data in relatives. Further forensic investigations including large families should be performed to decide the appropriate period time for a reanalysis of rare variants in SUDY/SIDS. The intervention of an experienced multidisciplinary team is highly recommended as well as the development of specific forensic guidelines to enable appropriate interpretation of rare genetic variants.

## Supplementary Information

Below is the link to the electronic supplementary material.

Supplementary file1 (PDF 390 KB)

## Data Availability

All data available in the manuscript.
