# Supplementary material for: Reevaluation of ambiguous genetic variants in sudden unexplained deaths of a young cohort
Source: Int J Legal Med. 2023 Jan 25;137(2):345–51. doi: 10.1007/s00414-023-02951-0 (PMC9902310; doi:10.1007/s00414-023-02951-0)
Supplement: Supplementary file 1 — Supplementary file1 (PDF 390 KB) [file 414_2023_2951_MOESM1_ESM.pdf]

Supplemental

Reevaluation of ambiguous genetic variants in sudden unexplained deaths of a young cohort

Suppl.1

Genetic analysis

A resequencing custom-made panel including the most prevalent genes associated with IAS and SIDS was used (*ABCC9, ACTA2, ACTC1, ACTN2, AKAP9, ANK2, BAG3, CACNA1C, CACNA1G, CACNA1H, CACNA1I, CACNB2, CASQ2, CAV3, CHRM2, COL3A1, CRYAB, CSRP3, CTF1, DES, DMD, DMPK, DSC2, DSG2, DSP, ECE1, EMD, EN1, EYA4, FBN1, FHL2, FKTN, GJA7, GLA, GPD1L, HCN1, HCN2, HCN4, ILK, JPH2, JUP, KCNA4, KCNA5, KCND2, KCND3, KCNE1, KCNE2, KCNE3, KCNH2, KCNJ2, KCNJ3, KCNJ5, KCNK4, KCNQ1, LAMA4, LAMP2, LDB3, LMNA, MYBPC3, MYH6, MYH7, MYL2, MYL3, MYLK2, MYOZ2, MYPN, NEBL, NEXN, NOS1AP, NOTCH1, NPPA, NUP155, PDLIM3, PHOX2A, PHOX2B, PKP2, PLN, PRKAG2, PSEN1, PSEN2, RBM20, RET, RYR2, SCN10A, SCN1B, SCN2B, SCN3B, SCN4B, SCN5A, SGCA, SGCB, SGCD, SIRT3, SLC25A4, SLC6A4, SLC8A1, SLMAP, SNTA1, TAZ, TCAP, TGFB3, TGFBR1, TGFBR2, TLX3, TMEM43, TMPO, TNNC1, TNNI3, TNNT2, TPM1, TTN,* and *VCL*). All gene isoforms are described in Ensembl 75 (www.ensembl.org/) linked to a RefSeq code (www.ncbi.nlm.nih.gov/refseq/) or CCDS (www.ncbi.nlm.nih.gov/CCDS/). Bioinformatic analysis included adaptor and low-quality base trimming of the FASTQ files. Trimmed reads were mapped with GEM III. The output was sorted, also uniquely and properly mapped read pairs were selected. Variant calling from the cleaned BAM files was performed with SAMtools v.1.2 and an *ad hoc*-developed script. The final annotation steps provided information included in public databases. Identified uncommon genetic variants, minor allele frequency (MAF) <1%, were confirmed by conventional Sanger sequencing. The exons and exon–intron boundaries of each gene were amplified in both directions.

All sequences were reanalyzed with the updated software (SeqScape v2.7, Applied Biosystems). No new rare variants were identified in any of the analyzed genes. The original classification compared rare variants with DNA sequences from 300 healthy Spanish individuals (individuals not related to any index case and of the same ethnicity) as control cases contrasted with the Genome Aggregation Database (gnomAD) (www.gnomad.broadinstitute.org/). Sequence variants were described following the Human Genome Variation Society (HGVS) rules (www.hgvs.org/). Currently, all rare missense variants were contrasted in the gnomAD (www.gnomad.broadinstitute.org/). All rare variants were also consulted in ClinGen (www.clinicalgenome.org/), VarSome (www.varsome.com/), the SCD-associated Variants Annotation Database (SVAD) (www.svad.mbc.nctu.edu.tw/), CardioClassifier (www.cardioclassifier.org/), InterVar (www.wintervar.wglab.org/), CardioVAI (www.cardiovai.engenome.com/), and CardioBoost (www.cardiodb.org/cardioboost/).

Suppl. 2

Data sources

Until June 2022, an exhaustive review of all available data on each rare variant was performed independently by three authors (EMB, GSB, OC), compared and verified. Data were collected from the Human Gene Mutation Database (HGMD) (www.hgmd.org), ClinVar (www.ncbi.nlm.nih.gov/clinvar/intro/), the National Center for Biotechnology Information single-nucleotide polymorphism (SNP) database (www.ncbi.nlm.nih.gov/SNP), Index Copernicus (www.en.indexcopernicus.com), Google Scholar (www.scholar.google.es), Springer Link (www.link.springer.com), Science Direct (www.sciencedirect.com), the Excerpta Medica Database (www.elsevier.com/solutions/embase-biomedical-research), and the IEEE Xplore Digital Library (www.ieeexplore.ieee.org/Xplore/home.jsp).

Tables legend (as Electronic Supplemental File)

(ESP_Tab1) **Table 1.- Genetic data of rare variants.** ACMG: American College of Medical Genetics and Genomics, B: benign, ClinVar: Clinical Variant, dbSNP: Single Nucleotide Polymorphism database, ExAC: Exome Aggregation Consortium, GnomAD: Genome Aggregation Database, LB: likely benign, LP: likely pathogenic, NA: not available, P: pathogenic, VUS: variant of uncertain significance.

(ESP_Tab2) **Table 2.- Cohort analyzed.**

Figures legend (as Electronic Supplemental File)

(ESP_Fig1) Figure 1.- Reclassification of rare variants. A.- All cohort. B.- All cohort concerning genes associated with channelopathies. C.- All cohort concerning genes associated with cardiomyopathies. D.- SIDS cohort. E.- SIDS cohort concerning genes associated with channelopathies. F.- SIDS cohort concerning genes associated with cardiomyopathies. G.- Young cohort. H.- Young cohort genes associated with channelopathies. I.- Young cohort genes associated with cardiomyopathies.

Tables

Table 1.- Genetic data of rare variants.

Case	Age	Sex	Situation of death	Macroscopic Heart	Microscopic Heart	Toxicology	Family History	Gene	Nucleotide	Protein	dbSNP	ExAC 2017	GnomAD 2022	ACMG 2017	ACMG 2022
1	<1 y	M	Sleeping	Normal	Normal	Negative	Negative	*FBN1*	c.6854A>G	p.Asp2285Gly	rs1160071679	NA	NA	VUS	VUS
2	<1 y	F	Sleeping	Normal	Normal	Negative	Negative	*RYR2*	c.5239G>A	p.Gly1747Ser	rs1553522190	NA	NA	VUS	VUS (LP)
3	<1 y	M	Sleeping	Normal	Normal	Negative	Negative	*RYR2*	c.505C>T	p.Arg169Ter	rs749930577	NA	0.0004	VUS	VUS (LP)
4	<1 y	M	Sleeping	Normal	Normal	Negative	Negative	*KCNJ5*	c.1123C>T	p.Arg375Trp	rs142454198	0.0041	0.0035	VUS	VUS
5	<1 y	F	Sleeping	Normal	Normal	Negative	Negative	*TTN*	c.58483G>C	p.Val19495Leu	rs768057735	NA	0.0064	VUS	VUS (LB)
6	<1 y	M	Sleeping	Normal	Normal	Negative	Negative	*TTN*	c.67781G>C	p.Ser22594Thr	rs750324994	NA	0.0012	VUS	VUS (LB)
7	<1 y	M	Sleeping	Normal	Normal	Negative	Negative	*MYH6*	c.4907G>A	p.Arg1636His	rs746448302	NA	0.0011	VUS	VUS (LB)
8	<1 y	F	Sleeping	Normal	Normal	Negative	Negative	*TTN*	c.49331C>G	p.Pro16444Arg	rs777291480	NA	0.0016	VUS	VUS (LB)
9	<1 y	M	Sleeping	Normal	Normal	Negative	Negative	*KCNH2*	c.874C>T	p.Arg292Cys	rs1196644896	NA	NA	VUS	VUS (LP)
10	<1 y	M	Sleeping	Normal	Slight fibrosis	Negative	Negative	*TTN*	c.76241T>C	p.Ile25414Thr	rs773976497	NA	NA	VUS	VUS
11	<1 y	M	Sleeping	Normal	Normal	Negative	Negative	*KCNH2*	c.1558-5C>T	NA	rs114186001	NA	0.16	VUS	B
12	<1 y	M	Sleeping	Normal	Normal	Negative	Negative	*TTN*	c.70546G>C	p.Val23516Leu	NA	NA	NA	VUS	VUS
13	<1 y	F	Sleeping	Normal	Normal	Negative	Negative	*EMD*	c.746A>G	p.Glu249Gly	rs781947413	NA	0.0049	VUS	VUS (LB)
14	<1 y	F	Sleeping	Normal	Normal	Negative	Negative	*DES*	c.1375G>A	p.Val459Ile	rs73991549	NA	0.249	VUS	B
15	<1 y	M	Sleeping	Normal	Normal	Negative	Negative	*NOTCH1*	c.7223T>C	p.Leu2408Pro	rs767728582	0.0017	0.0095	VUS	VUS (LB)
16	<1 y	F	Sleeping	Normal	Normal	Negative	Negative	*AKAP9*	c.6806A>G	p.Asp2269Gly	rs765553799	NA	0.0003	VUS	VUS (LP)
17	<1 y	M	Sleeping	Normal	Normal	Negative	Negative	*SCN10A*	c.1676A>T	p.Asn559Ile	NA	NA	NA	VUS	VUS (LP)
18	<1 y	M	Sleeping	Normal	Normal	Negative	Negative	*ANK2*	c.9439G>A	p.Val3147Met	NA	NA	NA	VUS	VUS (LP)
19	<1 y	F	Sleeping	Normal	Normal	Negative	Negative	*FBN1*	c.6161A>G	p.Gln2054Arg	NA	NA	NA	VUS	VUS
20	<1 y	F	Sleeping	Normal	Normal	Negative	Negative	*LAMA4*	c.3617A>G	p.Gln1206Arg	NA	NA	NA	VUS	VUS
21	<1 y	M	Sleeping	Normal	Normal	Negative	Negative	*MYLK2*	c.77G>A	p.Gly26Asp	rs765105352	0.0008	0.0008	VUS	VUS

22	<1 y	M	Sleeping	Normal	Normal	Negative	Negative	*DSP*	c.7153C>T	p.Arg2385Cys	rs755395467	0.0008	0.0003	VUS	VUS
23	<1 y	M	Sleeping	Normal	Normal	Negative	Negative	*DMPK*	c.535G>T	p.Ala179Ser	NA	NA	NA	VUS	VUS
24	<1 y	M	Sleeping	Normal	Normal	Negative	Negative	*MYH6*<br>*AKAP9*	c.2341G>T<br>c.7219G>C	p.Glu781Ter<br>p.Glu2407Gln	NA<br>NA	NA<br>NA	NA<br>NA	VUS<br>VUS	VUS<br>VUS (LP)
25	<1 y	F	Sleeping	Normal	Normal	Negative	Negative	*SLMAP*	c.829-5C>T	NA	rs1261534362	NA	0.0004	VUS	VUS
26	<1 y	M	Sleeping	Normal	Normal	Negative	Negative	*AKAP9*	c.6194T>C	p.Met2065Thr	NA	NA	NA	VUS	VUS (LP)
27	<1 y	M	Sleeping	Normal	Normal	Negative	Negative	*TTN*	c.84587C>A	p.Thr28196Lys	rs376537509	0.0008	0.0016	VUS	VUS (LB)
28	<1 y	F	Sleeping	Normal	Slight fibrosis	Negative	Negative	*NEBL*	c.1659A>T	p.Glu553Asp	rs779426326	0.0016	0.0016	VUS	VUS (LB)
29	<1 y	F	Sleeping	Normal	Normal	Negative	Negative	*TTN*	c.99566A>G	p.Tyr33189Cys	rs932225386	NA	NA	VUS	VUS
30	<1 y	M	Sleeping	Normal	Normal	Negative	Negative	*BAG3*	c.31C>G	p.Gln11Glu	NA	NA	NA	VUS	VUS
31	<1 y	F	Sleeping	Normal	Normal	Negative	Negative	*KCNA5*	c.640C>T	p.Arg214Cys	rs3197074	0.0008	0.0039	VUS	VUS
32	<1 y	F	Sleeping	Normal	Normal	Negative	Negative	*COL3A1*	c.3209C>G	p.Ala1070Gly	NA	NA	NA	VUS	VUS
33	<1 y	M	Sleeping	Normal	Normal	Negative	Negative	*SCN5A*	c.142G>A	p.Glu48Lys	rs199473048	0.0025	0.0052	VUS	VUS
34	<1 y	M	Sleeping	Normal	Slight fibrosis	Negative	Negative	*TTN*	c.63271G>A	p.Gly21091Ser	rs376256345	NA	0.0044	VUS	VUS (LB)
35	<1 y	F	Sleeping	Normal	Normal	Negative	Negative	*SCN5A*	c.5055G>C	p.Glu1685Asp	NA	NA	NA	VUS	VUS (LP)
36	<1 y	M	Sleeping	Normal	Normal	Negative	Negative	*KCNQ1*	c.1861G>A	p.Gly621Ser	rs199472820	NA	0.0051	VUS	VUS
37	<1 y	F	Sleeping	Normal	Normal	Negative	Negative	*AKAP9*	c.4927A>C	p.Ile1643Leu	rs141990258	0.0077	NA	VUS	VUS (LP)
38	2 y	F	Sleeping	Normal	Normal	Negative	Negative	*DSP*	c.916G>A	p.Ala306Thr	rs368193211	0.0077	0.0051	VUS	VUS (LB)
39	3 y	M	Emotion, stress, exercise	Normal	Slight fibrosis	Negative	Negative	*DSP*	c.314G>A	p.Arg105Gln	rs762238621	NA	0.0023	VUS	VUS (LB)
40	3 y	M	Sleeping	Normal	Normal	Negative	Negative	*TTN*<br>*TTN*	c.5577G>C<br>c.89494C>A	p.Arg1859Ser<br>p.Pro29832Thr	NA<br>rs373876117	NA<br>NA	NA<br>0.0119	VUS<br>VUS	VUS<br>LB
41	3 y	M	Sleeping	Normal	Normal	Negative	Negative	*TTN*	c.69373delATinsGA	p.Ile23125Asp	NA	NA	NA	VUS	VUS

42	10 y	M	Emotion, stress, exercise	Normal	Diffuse myocarditis	Negative	Negative	*MYH7*<br>*PKP2*	c.4772T>A<br>c.1237C>T	p.Leu1591Gln<br>p.Arg413Ter	rs61737004<br>rs372827156	NA<br>0.0077	0.0039<br>0.0011	VUS<br>VUS	VUS (LB)<br>VUS (LP)
43	10 y	M	Sleeping	Normal	Normal	Negative	Negative	*KCNH2*	c.2674C>T	p.Arg892Cys	rs201627778	0.0154	0.0442	VUS	LB
44	12 y	M	Sleeping	Normal	Normal	Negative	Negative	*GPD1L*	c.1009G>T	p.Val337Phe	NA	NA	NA	VUS	VUS
45	12 y	M	Emotion, stress, exercise	Normal	Normal	Negative	Negative	*RYR2*<br>*MYBPC3*<br>*PKP2*	c.217C>G<br>c.2398G>A<br>c.14G>A	p.Leu73Val<br>p.Gly800Arg<br>p.Gly5Asp	rs777753947<br>rs727504574<br>rs774778142	NA<br>NA<br>NA	NA<br>0.0031<br>0.0036	VUS<br>VUS<br>VUS	VUS (LP)<br>VUS (LB)<br>VUS (LB)
46	14 y	F	Emotion, stress, exercise	Normal	Normal	Negative	Negative	*ANK2*<br>*TGFB3*	c.7148C>T<br>c.755-5T>C	p.Pro2383Leu<br>NA	rs35960628<br>rs768188445	0.0308<br>NA	0.0119<br>0.0007	VUS<br>VUS	VUS (LB)<br>VUS
47	15 y	M	Emotion, stress, exercise	Normal	Normal	Negative	Negative	*ANK2*	c.7397G>A	p.Arg2466His	rs142078935	0.076	0.0714	VUS	LB
48	15 y	F	Emotion, stress, exercise	Normal	Normal	Negative	Negative	*MYH7*	c.2011C>T	p.Arg671Cys	rs727503263	NA	NA	VUS	VUS
49	16 y	M	Emotion, stress, exercise	Normal	Normal	Negative	Negative	*KCNH2*	c.526C>T	p.Arg176Trp	rs36210422	NA	0.0324	VUS	LB
50	16y	M	Emotion, stress, exercise	Normal	Normal	Negative	Negative	*RyR2*	c.32T>C	p.Ile11Ser	rs794728760	NA	NA	VUS	VUS (LP)
51	16 y	M	Emotion, stress, exercise	Normal	Slight fibrosis	Negative	Negative	*TNNI3*	c.611G>A	p.Arg204His	rs727504275	NA	NA	VUS	VUS

Table 2.- Cohort analyzed.

			< 1 year	1 – 16 years	Total
		**Cases**	37	14	51
**Males**	**Situation of death**	**Sleeping**	22	4	33
		**Emotion, stress, exercise**	0	7	
**Females**	**Situation of death**	**Sleeping**	15	1	18
		**Emotion, stress, exercise**	0	2	

Figures

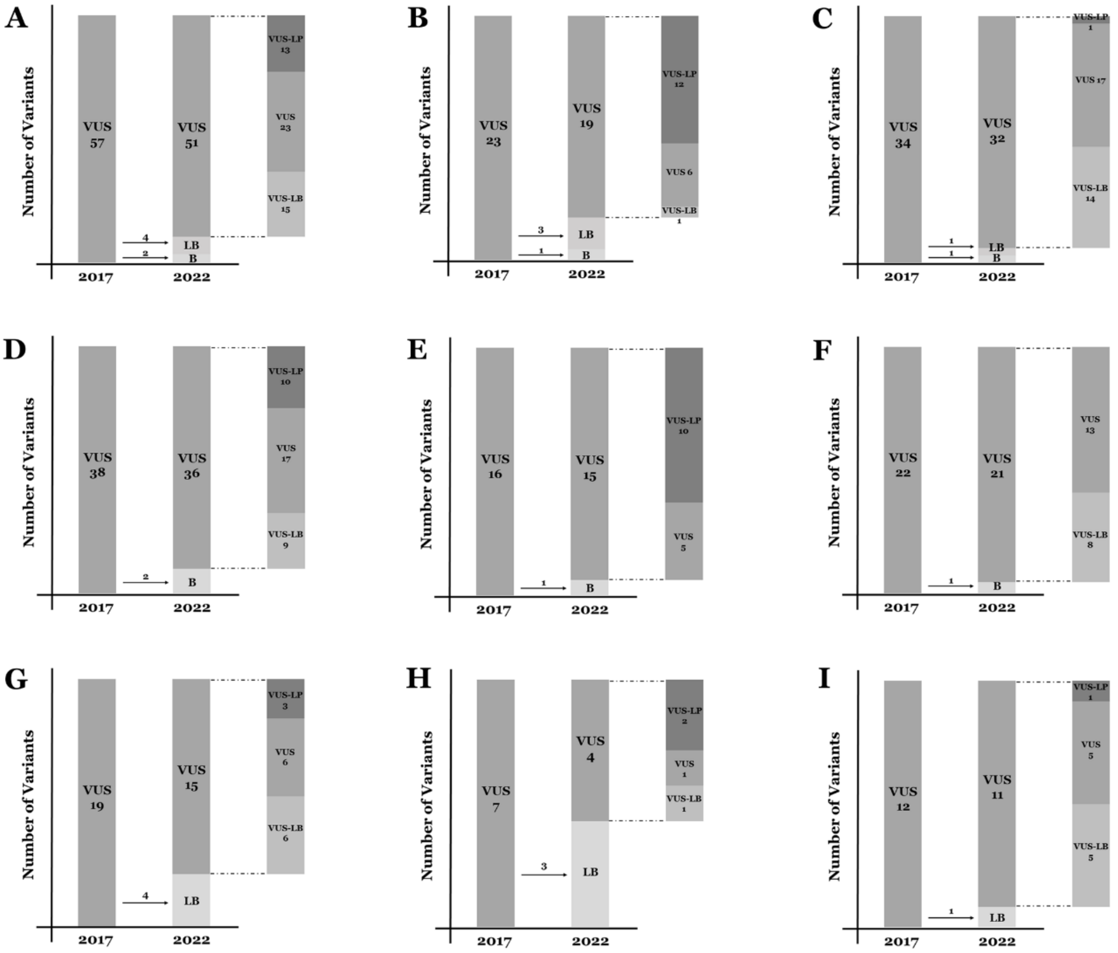
A
Number of Variants
VUS 57
VUS 51
4
LB
2
B
VUS-LP 13
VUS 23
VUS-LB 15
2017
2022
B
Number of Variants
VUS 23
VUS 19
3
LB
1
B
VUS-LP 12
VUS 6
VUS-LB 1
2017
2022
C
Number of Variants
VUS 34
VUS 32
1
LB
1
B
VUS-LP 1
VUS 17
VUS-LB 14
2017
2022
D
Number of Variants
VUS 38
VUS 36
2
B
VUS-LP 10
VUS 17
VUS-LB 9
2017
2022
E
Number of Variants
VUS 16
VUS 15
1
B
VUS-LP 10
VUS 5
2017
2022
F
Number of Variants
VUS 22
VUS 21
1
B
VUS 13
VUS-LB 8
2017
2022
G
Number of Variants
VUS 19
VUS 15
4
LB
VUS-LP 3
VUS 6
VUS-LB 6
2017
2022
H
Number of Variants
VUS 7
VUS 4
3
LB
VUS-LP 2
VUS 1
VUS-LB 1
2017
2022
I
Number of Variants
VUS 12
VUS 11
1
LB
VUS-LP 1
VUS 5
VUS-LB 5
2017
2022
